# Synthesis, Properties, Applications, and Future Prospective of Cellulose Nanocrystals

**DOI:** 10.3390/polym15204070

**Published:** 2023-10-12

**Authors:** Adib Bin Rashid, Md Enamul Hoque, Nahiyan Kabir, Fahim Ferdin Rifat, Hasin Ishrak, Abdulrahman Alqahtani, Muhammad E. H. Chowdhury

**Affiliations:** 1 Industrial and Production Engineering Department, Military Institute of Science and Technology (MIST), Dhaka 1216, Bangladesh; 2 Department of Biomedical Engineering, Military Institute of Science and Technology (MIST), Dhaka 1216, Bangladesh; 3 Department of Biomedical Technology, College of Applied Medical Sciences in Al-Kharj, Prince Sattam Bin Abdulaziz University, Al-Kharj 11942, Saudi Arabia; 4 Department of Medical Equipment Technology, College of Applied, Medical Science, Majmaah University, Majmaah City 11952, Saudi Arabia; 5 Department of Electrical Engineering, Qatar University, Doha 2713, Qatar

**Keywords:** cellulose nanocrystal, nanotechnology, physical adsorption, acoustic insulator, fire retardant

## Abstract

The exploration of nanocellulose has been aided by rapid nanotechnology and material science breakthroughs, resulting in their emergence as desired biomaterials. Nanocellulose has been thoroughly studied in various disciplines, including renewable energy, electronics, environment, food production, biomedicine, healthcare, and so on. Cellulose nanocrystal (CNC) is a part of the organic crystallization of macromolecular compounds found in bacteria’s capsular polysaccharides and plant fibers. Owing to numerous reactive chemical groups on its surface, physical adsorption, surface grating, and chemical vapor deposition can all be used to increase its performance, which is the key reason for its wide range of applications. Cellulose nanocrystals (CNCs) have much potential as suitable matrices and advanced materials, and they have been utilized so far, both in terms of modifying and inventing uses for them. This work reviews CNC’s synthesis, properties and various industrial applications. This review has also discussed the widespread applications of CNC as sensor, acoustic insulator, and fire retardant material.

## 1. Introduction

Cellulose, the most abundant natural polymer on Earth, has garnered significant attention in recent years for its transformation into nanocrystals, known as cellulose nanocrystals (CNCs). CNCs, with their exceptional mechanical, thermal, and optical properties, have emerged as a versatile and sustainable nanomaterial with the potential to revolutionize various industries [1]. 

The roles of cellulose nanocrystals’ (CNC) nonlinear geometry in improved performance coatings are appealing for mini structures or device production; yet, CNC is an insulating material. The cellulose nanocrystal (CNC) is a part of the organic crystallization macromolecular compound found in plant fibers and bacteria’s capsular polysaccharides. It has several properties, which include high strength, high crystallization, large physical properties, minimum density, and good biocompatibility [2].

CNC has a minimal density, specific impact resistance, low thermal expansion, superior stiffness, elongated geometry, barrier properties, an elevated surface area and relative density, and bio-conjugation availability and simplicity. Because of the numerous reactive chemical groups on its surface, chemical vapor deposition, physical adsorption, and surface grafting can all be used to enhance its performance. CNC research has received much interest in the last few years, as seen by the significant rise in academic papers and patents published worldwide. As a result, it is extensively applied in industries including construction, cuisine, telecommunications, medication, and barriers [3]. Carbon nanotubes (CNTs) are another rod-shaped nanomaterial with excellent mechanical and electrical conductivity performance. 

Cellulose, when combined with the functionality of reduced graphene oxide, has unique advantages. Kafy and his associates concluded that graphene oxide improves the electronic communication between enzymes [4]. Graphene oxide measures the alterations in the electric conductivity of the material. Graphene also possesses unique surface features, such as the ability to modify its surface. The advantage of graphene is that it is a very low-noise material. As a result, graphene’s carrier concentration can alter dramatically even with no carriers and a few more electrons. In this application, graphene also enables the generation of four-probe devices on monocrystals, which is an added benefit. This ensures that the contact resistance’s role in limiting sensitivity is no longer a factor [5]. 

Nanocellulose has been used to enhance materials’ physical and dispersibility attributes because of its high stiffness, special water dissolution rate, and hydrophilic aspects. Hydrolyzed polymeric cellulose surface morphology reduces obstacles to more significant movement between the analyte and the immobilized indicator, resulting in a faster response time [6]. The cellulose nanocrystal film is an attractive fit for wearable sensors because of the high energy engagement between the binding site on the nanocrystalline cellulose film and the charged species and the membrane separation properties [7]. CNC stands out among other nanostructured materials as a biocompatible, nontoxic, sustainable, and renewable nanomaterial. CNC has numerous potential uses in multiple fields, for instance, materials science, electronics, medicine, and many more, owing to its nanometric size, huge aspect ratio, and superior chemical and mechanical characteristics.

In essence, this review paper aims to provide a thorough understanding of cellulose nanocrystals, from their synthesis to their multifaceted applications, and to highlight their potential as a sustainable and innovative nanomaterial for the future. 

## 2. Synthesis of CNC

Cellulose is abundant in plants and microbes and significantly impacts the synthesis of macrostructure polymer materials. Cellulose nanocrystal has shown their importance in various uses in the industry due to their excellent material characteristics [3]. Cellulose nanocrystals are distinct nanoparticles generated from the most prevalent and seemingly natural polymer, inexhaustible cellulose. These nanostructures’ chemical, rheological, optical, and mechanical properties have attracted people’s interest. Cellulose nanocrystals, often generated from pre-existing cellulose fibers, are biodegradable and renewable, making them ideal for various uses [8]. Although naturally hydrophobic, these nanocrystals can be surface-functionalized to meet multiple demanding requirements, including creating high-performance nanocomposites with hydrophobic polymer matrixes [9]. This review attempts to consolidate knowledge concerning cellulose nanocrystal sources, chemical composition, chemical isolation procedures, and physical and rheological, mechanical, and optical properties considering the growing interdisciplinary research being conducted on them. Innovative applications of cellulose nanocrystals in domains as diverse as bioengineering, material sciences, semiconductors, catalysis, and various others are emphasized [10].

**Figure 1:**
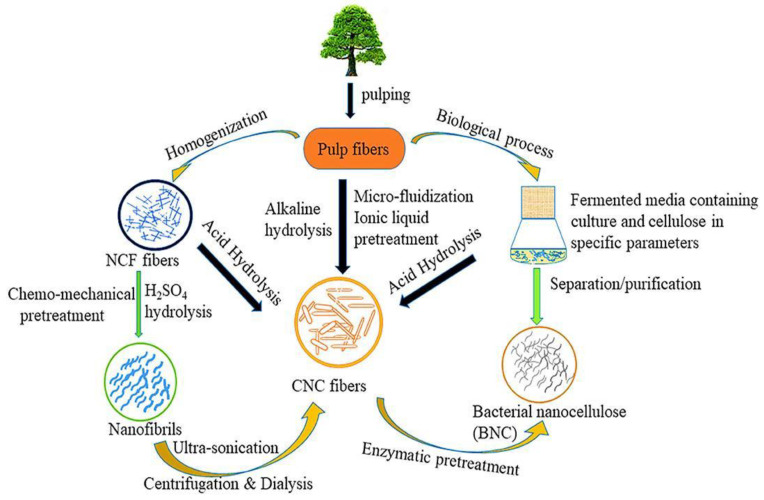
Production process of nanocellulose [12].

The preparation processes (Figure 1) of the CNC were evaluated in this review as sustainable future material. Meanwhile, brief introductions were given to CNC’s significant applications in composites, energy consumption, semiconductors, and barrier film. The comprehensive preparations and applications yielded practical concepts and methodologies for future high-end and environmentally friendly functional composites. There were also some suggestions for extended probable applications [11].

Plant fibers are commonly used in preparing CNCs due to their inexpensive cost and abundance, with a standard length and diameter. Meanwhile, the CNC refers to the size of any one-dimensional cellulose located in the range of 100 nm [13]. As a result, the processes for obtaining the CNC reduce the size of natural cellulose, which include mechanical, chemical, biological, and combination. 

### 2.1. Mechanical Process of Preparation of CNC 

**Figure 2:**
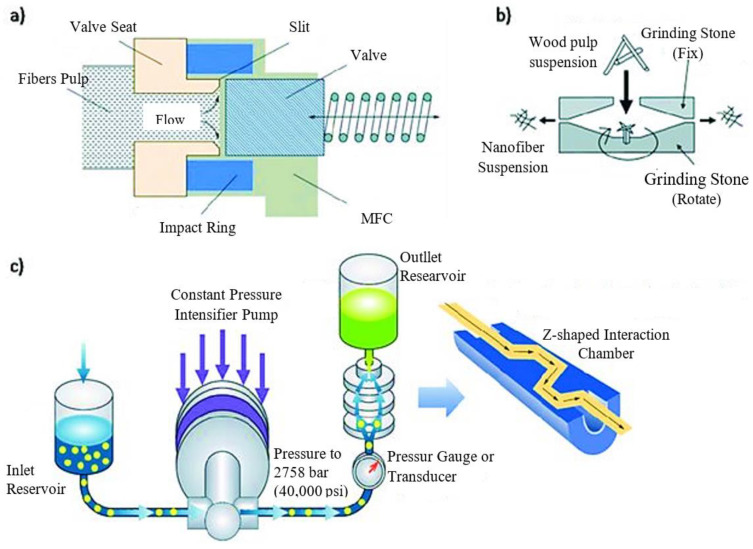
Operation schemes of (**a**) high-pressure homogenizer; (**b**) micro grinder from Masuko Sangyo Co.; and (**c**) micro fluidizer from Microfluidics Inc. [14].

The mechanical process (Figure 2) includes four steps and is a physical approach to obtaining the CNC:

(i) Homogenization under high pressure;
(ii) Micro fluidization;
(iii) Fine grinding;
(iv) Freezing smashing.

#### 2.1.1. Homogenization under High Pressure

It takes just one step to homogenize a solid into ultrafine particles in a solution, which results in a stable suspension. High-pressure homogenization was the most common method for producing microcrystalline cellulose (MCC). Several investigations have shown that it can be used to make MCC from various basic materials [14].

#### 2.1.2. Micro Fluidization

**Figure 3:**
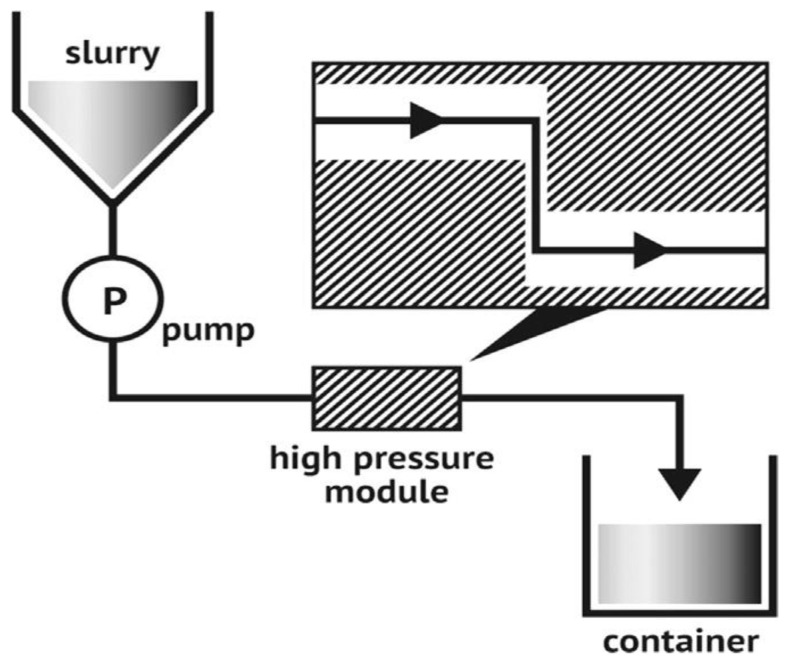
A schematic diagram of the micro fluidization process [16].

Figure 3 depicts the technical process of micro fluidization under high pressure, one way of creating nanomaterial. The fluid pump initially injected and compressed the raw slurry to a pressure of roughly 4000 bars. After that, it was thrown into a Y-shaped interactive tank, where two liquid squirts traveling at 1000 m/s collided. The vacuum effect and enormous shear force generated by this impact lead to the production of nanoparticles [15].

#### 2.1.3. Fine Grinding

**Figure 4:**
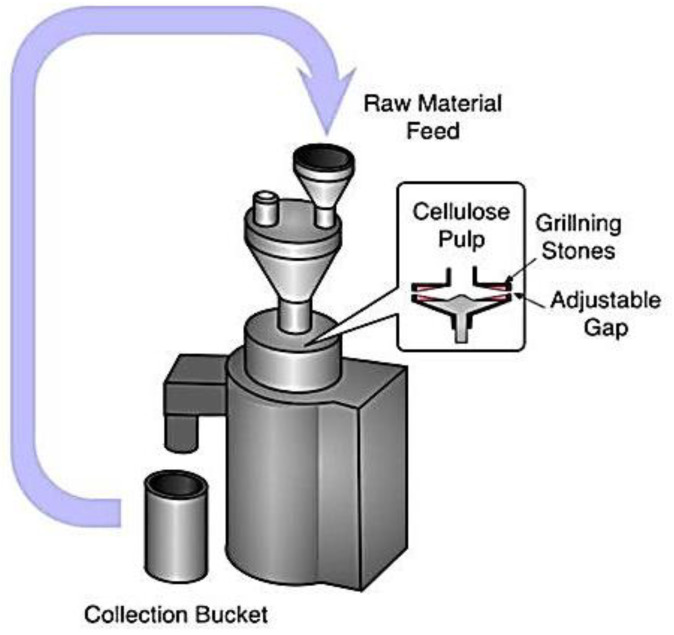
Ultra-friction grinder for the synthesis of cellulose nanofiber (CNF) [15].

The fine grinder and a commercial grinder worked together to provide a new physical approach to making MCC. It was clear that the main components were made up of two discs, one internal and one exterior, each covered with grooves with a unique set of characteristics. As the two discs rotated in tandem, they applied crushing, shearing, friction, grinding, and ripping forces that caused the fibers to split and shrink. However, it was very inefficient, and only a few instances of its use were documented [15]. In Figure 4, we can see the experimental setup for fine grinding. The raw material is poured into the funnel, and, inside the chamber, the cellulose pulp passes through grilling stones and an adjustable gap. Finally, the finished product is collected from the collection bucket.

### 2.2. Chemical Process

The CNC can only be obtained by partly disrupting glycosidic bonds; chemical hydrolysis makes a strong argument for attaining this. A kind of polysaccharide, cellulose, is created from glucose molecules joined together by the β-1,4-glucosidic link [17].

#### 2.2.1. Alkali Hydrolysis

Natural cellulose’s crystal shape changed from I to II after being subjected to a 9-weight-percent NaOH treatment. The alkali has the power to both expand the cellulose and rupture its internal hydrogen bonds. Even though alkali has been used to hydrolyze cellulose in several studies, alkali was mainly used to dissolve lignin and pectin [18].

#### 2.2.2. Acid Hydrolysis

**Figure 5:**
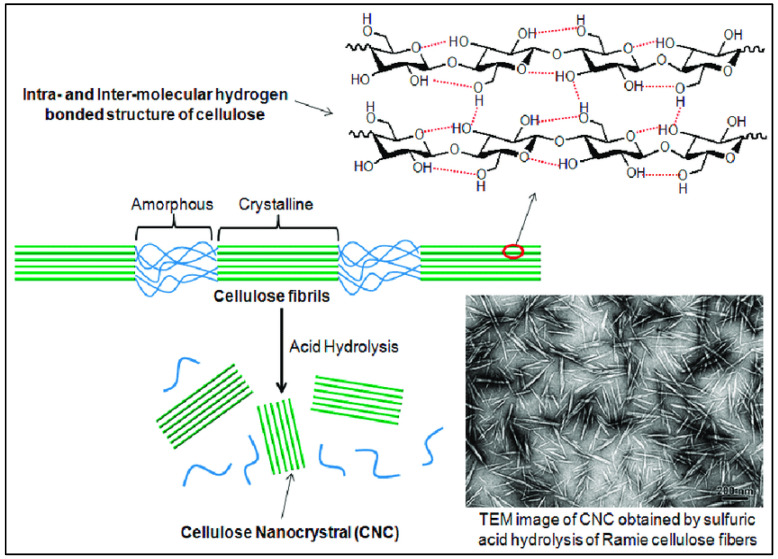
Manufacturing of CNC by acid hydrolysis and the corresponding TEM image [20].

Repeating the above structures yielded the cellulose fiber, composed of discontinuous sections of crystalline and amorphous cellulose. The CNC was made from reed pulp utilizing sodium m-nitrobenzene sulfonate (SMS) as a cocatalyst and 55-weight-percent sulfuric acid. The final CNC had an I-form and a rod-like shape, measuring 12 nm in diameter and 146 nm in length. Its crystallinity was 76.1 percent. On the other hand, the CNC-produced thermal insulation foam demonstrated its flawless thermal insulation ability under typical conditions. The ideal time range for the hydrolysis of this fiber was discovered to be between 45 and 55 min [19]. We can see the internal structure of cellulose (Figure 5), where two types of regions are present. An unorganized blue portion is called the amorphous region, and the green liner pattern is called a crystalline region. To obtain CNC from the amorphous cellulose region, concentrated 98% sulfuric acid must be broken.

## 3. Properties of CNC

**Figure 6:**
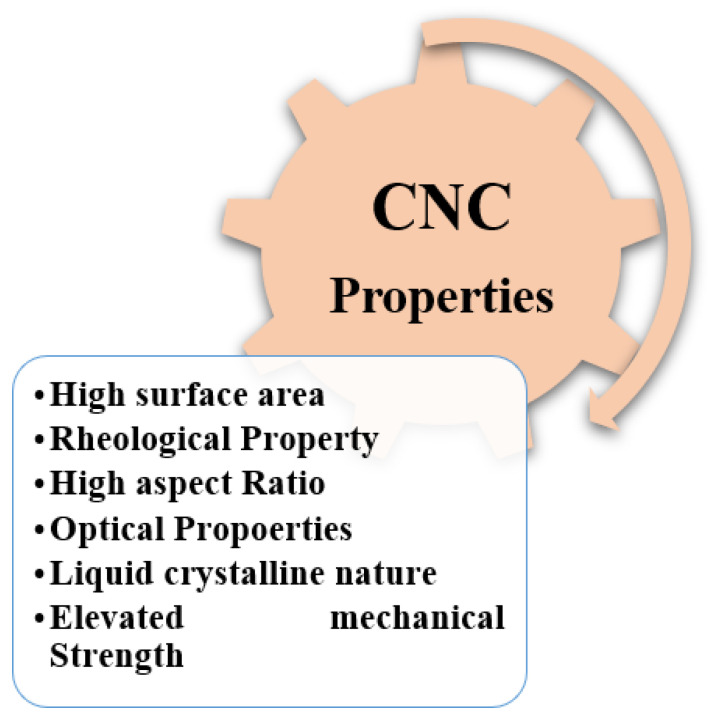
Properties of CNC.

CNCs have high mechanical strength, a large surface area, an elevated aspect ratio, liquid crystalline nature, thermal properties, rheological qualities, optical properties, and many others (Figure 6).

### 3.1. Mechanical Properties

Cellulose nanocrystals (CNCs) are promising recyclable nanomaterials with excellent mechanical qualities that can be used in various applications. According to research, CNCs can be identified into bundles, and their theoretical tensile strength is substantially higher than steel wire. The elastic modulus and the transverse elasticity of CNC are tested using the bending test. CNC has a high elastic modulus and a transverse modulus value [21].

**Figure 7:**
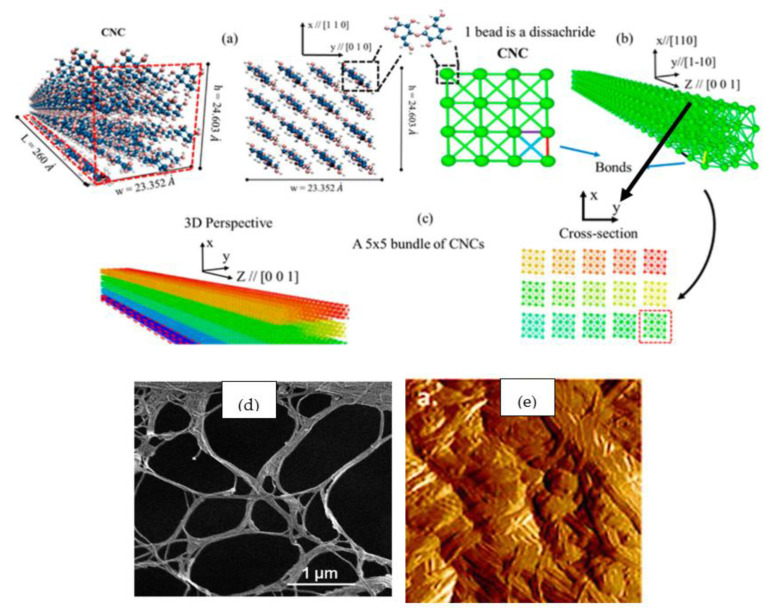
The three-dimensional atomic structure of cellulose 3D nanocrystals (**a**–**c**) [22]. Scanning electron microscopy (SEM) image of nanocellulose (NC) (**d**) [23]; atomic force microscopy (AFM) images (peak-force error) of NC (**e**) [23].

In Figure 7a, the atomic structure of cellulose nanocrystals is depicted in three dimensions (3D), where blue spheres signify carbon atoms, pink spheres symbolize oxygen atoms, and white spheres signify hydrogen atoms. Figure 7b shows a three-dimensional and cross-sectional picture of a five-CNC bundle with various particles colored differently [22]. Figure 7d,e shows the SEM image of dried NC and AFM image NC films, respectively.

### 3.2. Thermal Properties

Cellulose nanocrystals (CNC) are nature’s most abundantly available biopolymer. Due to their widespread availability and unique characteristics, CNC has been put into many different applications [24,25]. It has a lower density, high crystallinity, tensile strength, and modulus, which could be made in huge quantities at a minimal cost [26,27].

**Figure 8:**
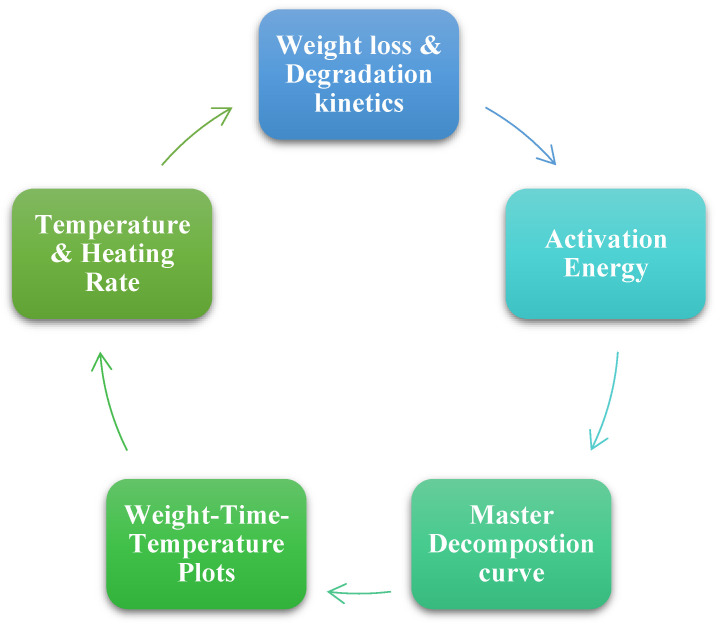
Thermal properties of CNC.

The high crystallinity index and mechanical qualities of nanocellulose may influence thermal parameters such as thermal capacity and expansion and, as a result, the use of nanocellulose in electrical devices and packaging is increasing. The lower thermal stability of nanocellulose can limit its application for nanocomposite manufacture at high temperatures. As a result, defining the high service temperature for the products required and consumption requires a thorough analysis of the thermal properties. As a result, this review examines the literature on the thermal characteristics and measurements of nanocellulose and its nanocomposites. This paper aims to call attention to recent research on the influence of CNC on nanocomposites’ thermal properties and its extensive applications in various sectors. CNC was studied for its thermal degradation, thermal transitions, thermal conductivity, and thermal expansion (Figure 8) [28].

### 3.3. Liquid Crystallinity

The nematic phase causes asymmetrical rod-like or plate-like nanoparticles to spontaneously form structured patterns in the right conditions and at crucial concentrations. When chiral nematic stages with liquid crystalline characteristics are distributed in water, it is seen that, to synthesize chiral nematic phases, rod-like CNCs self-align. Because of their rigidity, aspect ratios, and ability to align under precise conditions, they are perfect for exhibiting liquid crystalline behavior. On either hand, viscose crystallites have a helical twist down a long axis, like a screw, which can result in chiral nematic or even cholesteric phases of stacked surfaces aligned across a perpendicular axis, depending on the concentration [29].

Various aspects, including dispersity, shape, charge, size, electrolyte, and external stimuli, can modify CNC’s liquid crystallinity. The birefringent characteristic of nanocrystals and their liquid crystallinity results in several fascinating optical phenomena [26].

The crystalline nature of the liquid can also be influenced by the type of acid employed for hydrolysis. CNC produced from the hydrolysis of sulfuric acid frequently has a negatively charged surface, which aids uniform diffusion into the water due to the electrostatic coulomb repulsion. Despite the strong contacts between nanocrystals, heavily sulfonated CNC is easily dispersed, resulting in lyotropic behavior. CNCs formed from sulfuric and phosphoric acids often have chiral nematic structures, whereas CNCs derived from hydrochloric acid with postreaction sulfonation have a birefringent glassy phase [30].

### 3.4. Rheological Properties

For example, liquid crystallinity, order, and gelation quality determine CNC rheological features. Because it comprises rod-shaped nanocrystals, the CNC shows shear thinning performance at low concentrations and anomalous behavior at greater attention when the suspensions are lyotropic [31].

CNC suspension is made out of a nematic framework. Additionally, as the relaxation constant is proportional to the aspect ratio, CNCs with larger aspect ratios stay lined up for longer after shear. The rheological properties of CNC suspensions can also be affected by the type of solution used to hydrolyze them [9].

Numerous studies are currently looking into the rheological characteristics of these celluloses by attempting to explain how their microstructure changes during processing, handling, and application. Nanofibers of cellulose with a high aspect ratio makes up cellulose nanocrystals (CNC), which have distinct rheological properties. Above a critical concentration, the CNC aqueous solution displays a liquid crystal (LCs) state because of the rod-like structure and surface charge of the particles. The CNC suspensions made by concentrated sulfuric acid hydrolysis are typically isotropic up to 3 weight percent, phase-separate into liquid crystalline and isotropic biphasic suspensions at higher concentrations, where the viscosity profile exhibits a typical three-region behavior and behave as rheological gels that exhibit single shear thinning behavior at even higher concentrations [32].

The rheological characteristics of CNC suspensions can be considerably influenced by temperature. The change in the relative proportions of isotropic and liquid crystalline areas is thought to be the cause of this temperature dependence on the rheological properties. Jia also examined the rheological characteristics of an amorphous cellulose suspension and talked about how concentration, ionic strength, pH, and temperature affect the flow and viscoelastic characteristics of amorphous cellulose suspensions. The study of the rheological characteristics of CNC suspensions has received a lot of interest, but it is still difficult to fully grasp how the microstructure of these suspensions affects their rheological characteristics [32,33].

### 3.5. Optical Properties

CNC has much potential in fabricating bio-inspired materials with sophisticated features. The conversions from liquid crystal suspensions to long-range order inside the dry state are crucial for the select mechanical characteristics of CNC-made components. Until now, related transitions have only been investigated utilizing simple interfaces such as planar substrates. As they grow onto mesh supports, such changes are investigated. For the creation of CNC films, the meshed substrate provides a complicated topology similar to that found in nature. Self-assembly of the CNCs takes place in confinement and supports a framework that surrounds the mesh holes. This results in nematic and/or asymmetric nematic order in coexisting suspended and reinforced nanoparticle layers. The production of the suspending films happens by intermediate gel formation or kinetic suspension of CNCs from across the mesh’s open sections, according to optical microscopy with crossed polarizers. The suggested simple and scalable approach produces self-standing, ultrathin CNC films with adjustable optical properties, such as selected reflections in the visual spectrum (structural color) [26].

**Figure 9:**
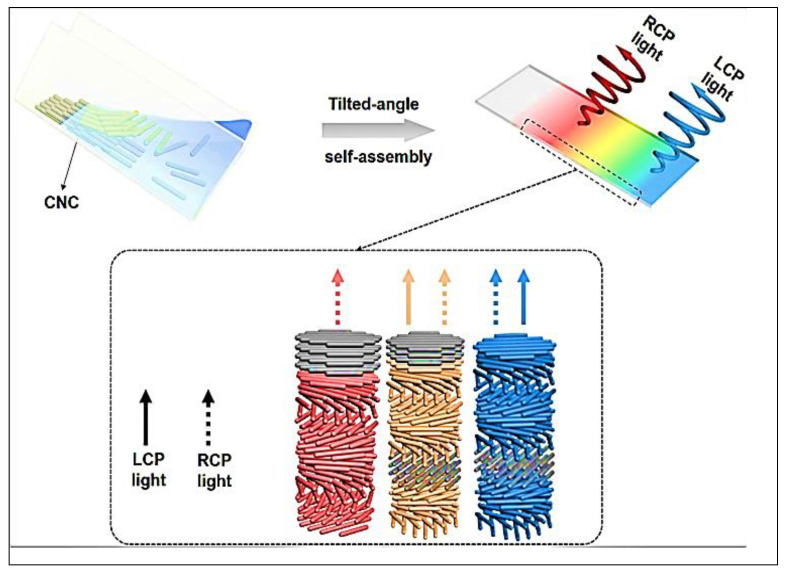
Self-organized CNC films with rainbow hues and ambidextrous optical reflection, produced via tilted-angle self-assembly method [34].

Self-assembly at a slightly curved angle provides self-organized CNC films with vibrant color and double-fronted optical reflection, as shown in Figure 9. LCP light is formed when a left-handed chiral nematic structure actively reflects incident light. Embedded nematic-like and left-handed PBG layers produce RCP light at the film–substrate interface, which reverts transmitted RCP light in situ. In situ handedness reversal of LCP light to RCP light is caused by the nematic-like layer implanted within two identical left-handed PBG layers. The nematic-like barrier converts RCP light directly to LCP light, which is eventually absorbed by the left-handed PBG layer below and retreats to RCP light when it passes through the nematic-like barrier. The overall reflectance of a mesostructured feature exceeds the 50 percent limit of a left-handed asymmetrical nematic arrangement. Gray denotes a nematic-like structure; solid arrows represent LCP light, whereas dotted arrows represent RCP light [34].

### 3.6. Fire Retardant Material

One of the earliest methods of finishing textiles is the application of flame retardants; risks related to the easy combustibility of cellulosic materials were understood as early as the 4th century B.C. Using a solution of ammonium phosphate, ammonium chloride, and borax to treat hemp, jute, and linen fabric in 1821 in France gave rise to the idea of preventing textiles from burning. The work of Perkin, who precipitated stannic oxide into the fiber, served as the foundation for the first practical, washable, flame-resistant treatment for cloth. Despite being flame-resistant, the fabric was entirely consumed by the afterglow, which was strong and persistent [35].

**Figure 10:**
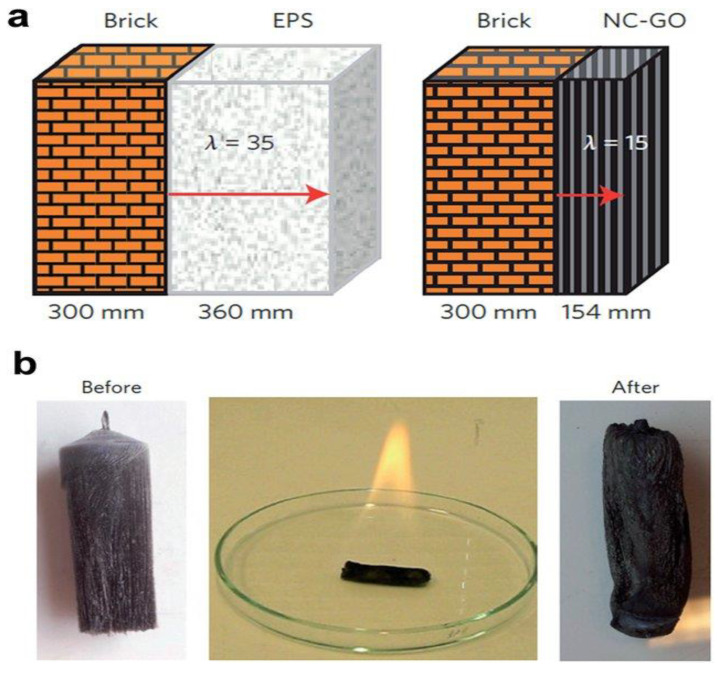
(**a**) Nano-cellulose-based composite foam with sepiolite (SEP), graphene oxide (GO), and boric acid (**b**) offers thermal insulation and fire resistance [38].

CNC works as a fire retardant (Figure 10) material as it is a hydrophilic compound. Cellulose insulation helps to keep homes safer by giving up to 50% more survivability than fiberglass insulation. In practice, this implies that inhabitants will have more chances to go to safety in the event of a fire [36,37].

Low fire resistance is a property of cellulosic textiles. They quickly burn because they are made of the fuels carbon and hydrogen as well as the combustion supporter oxygen. Cellulosic materials undergo oxidation during the burning process. This process may be accompanied by a flame or light; when the flame has been put out, most organic fibers glow. Since the glow can totally consume a textile, it may be just as damaging as the actual flame. The mechanisms of flaming and glowing are very different from one another and occur at various temperatures [39]. Before delving into the specifics of how to finish a textile such that it is fire and flame-retardant, it is helpful to establish a few terminologies [37,40].

## 4. Applications of Cellulose Nanocomposite

CNCs are a particular type of nanomaterial because of their large surface area and area ratio and the presence of functional groups. Nanoscale CNCs have specific surface areas ranging from one hundred meters square to several hundred meters square. The enormous surface area of CNCs facilitates interfacial interactions with other polymers. The higher the surface-to-volume ratio between CNCs and their surroundings, the stronger the interaction. The surface area increased because of this. As a result of interaction matrices, CNC dispersion in polymeric materials improves. As a result, the CNC distribution becomes more uniform. Their effectiveness is crucial when it comes to the desired qualities of CNCs. In nanocomposites, lower concentrations are attained [41].

The wide applications of nanocellulose are linked to its unique features such as nontoxicity, high strength and modulus, lightweight, thermal stability, dimensional stability, high thermal conductivity, low oxygen permeability, high optical transparency, mild abrasiveness, and the fact that it is recyclable, reusable, biodegradable, and environmentally friendly [42].

Table 1 and Table 2 highlight numerous prospective markets for nanocellulose-based materials, including plastics, high-volume operations in the automotive, pulp and paper, and food industries, as well as low-volume applications in cosmetic, medicine, and pharmaceutical sectors (e.g., biomedical implants and drug delivery) and building sectors (e.g., barrier/coating and strength additions in nanocomposites). Among these are photovoltaics, photonic films, 3D printing, and recyclable electronics, to mention a few [42].

### 4.1. Sensing Applications

Because of highly reactive functional groups, CNCs can be attached to various biological components and metallic nanoparticles for biosensors and biomedical imaging applications. CNCs are exceedingly hydrophilic, making them inappropriate with hydrophobic polymer matrices, and they have low electrical conductivities, despite their many advantages. As a result, numerous ways for modifying CNCs to gain appropriate functions as reference material in sensor fabrication have been used. Even though CNCs are the most widely observed nanocrystal type, other types of NCs should be incorporated to complete the picture. This review paper discusses the recent potential applications of nanocrystals in sensor fabrication. It discusses how nanocrystals could detect gases, chemicals, proteins, and other biomolecules. Due to their unique attributes, CNCs’ synthesis, properties, and applications are all heavily emphasized [41] (Table 3).

#### 4.1.1. Gas Sensing

Timely identifying hazardous gases such as NO_2_ could help effectively treat their detrimental consequences. On the other hand, effective detection of ammonia and ethylene might drastically reduce waste. Colorimetric and electrochemical sensors have been utilized in several sensing applications, alone or in collaboration with other technologies. The chemical reaction of gas molecules with the active compounds of the sensors results in an electrical conductivity change in the sensors, which is the basis for electrochemical sensing. The produced electrical signals are pretty similar to the desired gas concentrations [28,71].

PANI/CNC composite samples’ electrical resistance was affected when subjected to ammonia due to PANI deprotonation. In less than 10 s, the composites responded to ammonia. Furthermore, due to the huge surface area of CNCs, this detector was highly sensitive to ammonia, with a detection limit of 10 ppm [72,73].

**Figure 11:**
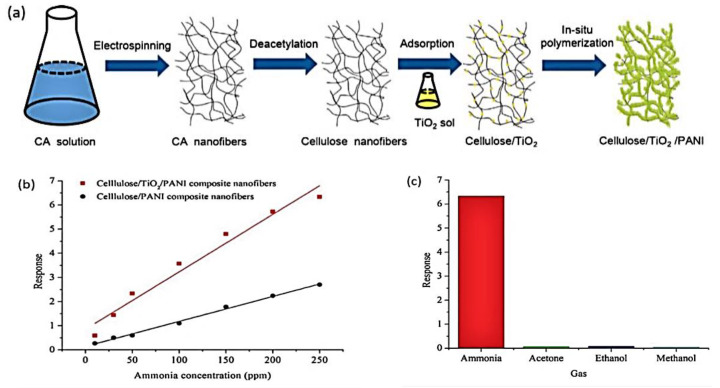
(**a**) Cellulose/TiO_2_/PANI system is depicted. (**b**) Cellulose/PANI and cellulose/TiO_2_/PANI respond to several ammonia concentrations (10–250 ppm). (**c**) The selectivity of cellulose/TiO_2_/PANI in various solvents [71].

Figure 11a depicts an ammonia gas sensor at room temperature that was constructed using cellulose, titanium oxide (TiO_2_), and PANI (cellulose/TiO_2_/PANI). The findings revealed that exposing the ammonia composite could cause a change in its electrical resistance. The material became more conductive after being doped with HCl. However, as the HCl-treated cellulose/TiO_2_/PANI absorbed ammonia, it was re-doped, making it less resistant. After ammonia desorption, the composite’s resistance fell once more. Furthermore, due to creating a P–N junction between TiO_2_ and PANI, cellulose/TiO_2_/ PANI composites demonstrated a more incredible reaction to ammonia than cellulose/PANI alone (Figure 11b). It was also discovered that the composite’s sensitivity to ammonia was substantially more significant than that of other gases, including acetone, ethanol, and methanol, as shown in Figure 11c [41].

#### 4.1.2. Chemical Sensors

Some cellulose compounds, such as cellulose acetate, nitrate, and carboxymethyl cellulose, have numerous functional groups, making them great functionalization matrices. Enzyme-adsorbed programs can detect a large array of compounds at low levels [41]. When analytes (chemical stimuli) interact with the sensing layer, they change some physical properties (conductivity, temperature, work function, mass, and refractive index), which the transducer then converts into a variation in the corresponding electric factor, resulting in the final sensor signal [74,75].

**Figure 12:**
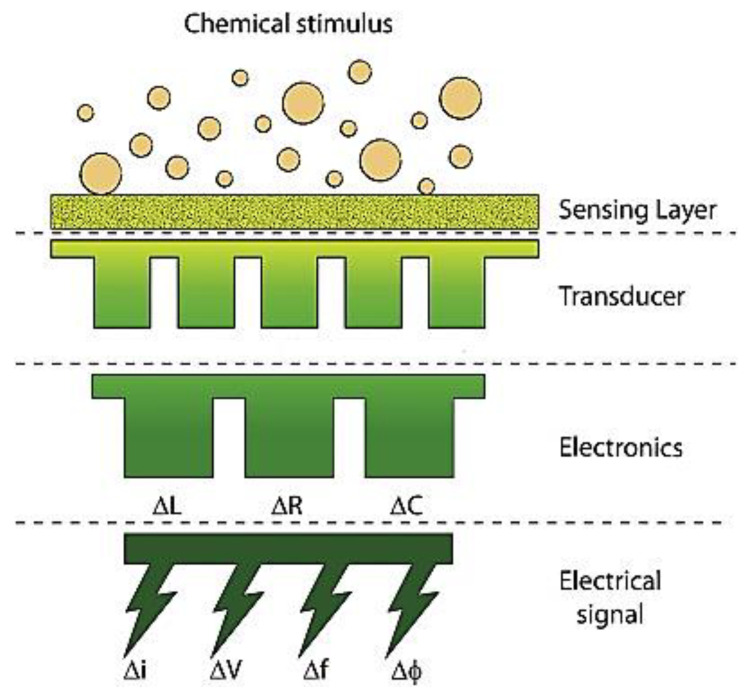
A chemical sensor’s schematic structure [75].

A chemical sensor’s schematic structure is shown in Figure 12. The sensing layer changes its physical properties (Δ*n*: refractive index, Δ*m*: mass, Δ*Φ*: work function, Δ*T*: temperature, Δ*σ*: conductivity) in response to analytes (chemical stimulus), which are then converted by the transducer into a variation in the corresponding electric parameter, resulting in the generation of the final sensor signal [75].

#### 4.1.3. Protein Detector

**Figure 13:**
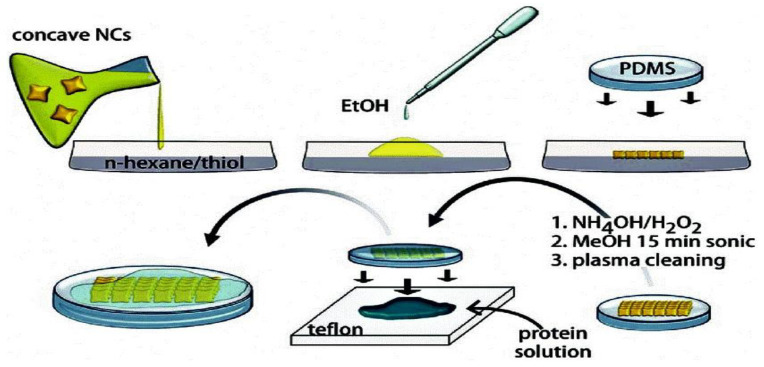
The construction of the CNC arrays [11].

A biosensor’s ability to spot enzymes in low amounts is essential in many uses, particularly in the biomedical industry [76]. Because enzyme quantities vary widely depending on pathological conditions, enzymes could be utilized as a disease marker. The tripeptide/CNC sensors responded to HNE 10–20 times faster than the tripeptide/paper sensors. This is because CNCs have 8000 times the surface area of cellulosic paper, with 131 and 0.0226 m^2^ gL, respectively. Due to the high surface area of such sensors, more elastase was obtainable for interaction with HNE, resulting in increased sensitivity [77]. The schematic diagram of CNC arrays’ production, immobilization over polydimethysiloxane (PDMS) films, and the processing of protein samples for SERS analysis is given in Figure 13.

#### 4.1.4. Humidity Sensing Measurement

UPS thought CNCs were viable for employing capacitive micromachined ultrasonic transducers (CMUT) as a humidity-sensing sensor. This was confirmed by humidity sensing data over an RH range of 11% to 94% [4]. When the CMUT and CNC sensing films were combined, they demonstrated superior RH sensitivity of up to 2 kHz, 2.5 percent RH hysteresis, remarkable repeatability, and stability for a long time. While the CMUT humidity sensor exceeded its competitors in terms of overall humidity identification, the CMUT design and CNC performance characteristics might be improved even further to maximize RH sensitivity. The diameter of the CMUT membranes can be lowered to optimize the resonance frequency and mass sensitivity. In terms of functionalization, increasing the thickness of the CNC film causes more water molecules to be captured, resulting in improved sensitivity [69,78].

**Figure 14:**
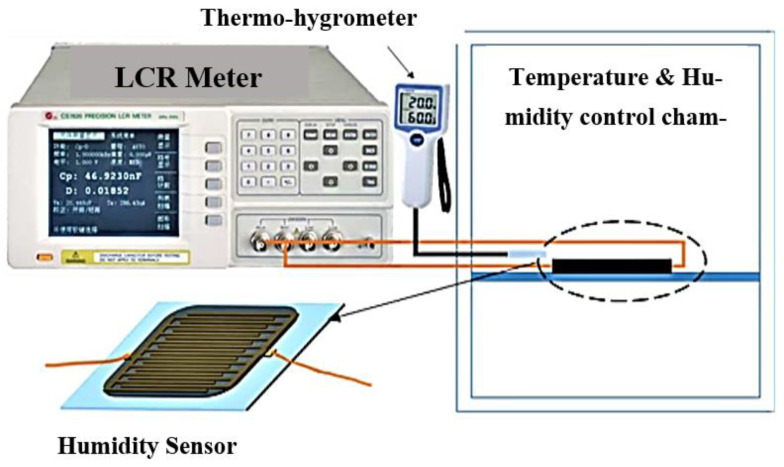
The constructed sensor and its performance measurement are depicted in the schematic diagram [4].

An interdigital transducer (IDT) patterned electrode was deposited on a PET substrate using a typical lift-off process to make the humidity sensor. Drop by drop, the CNC/GO solution was poured onto the pattered electrode and dried in a 60 °C oven. The IDT patterned electrode’s two ends were then linked together and attached to an LCR (inductance, capacitance, and resistance) meter (HP 4284A). The schematic of the constructed sample is shown in Figure 14. The IDT electrode has a comb distance of 27 m [4].

### 4.2. Industrial Applications

**Figure 15:**
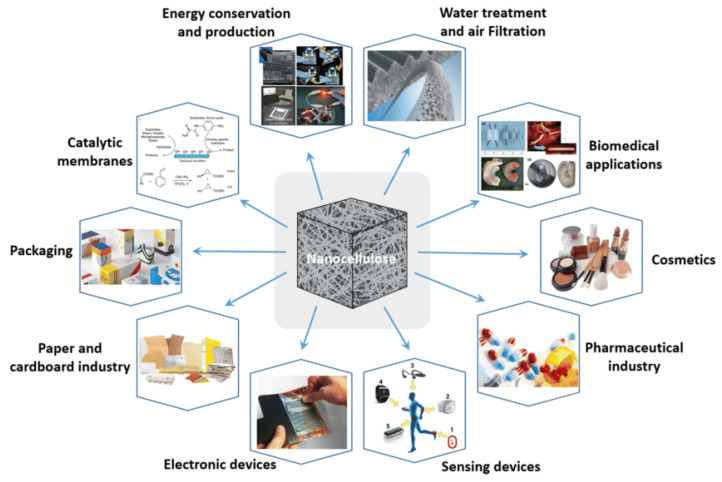
State-of-the-art applications for new nanocellulose-based materials [80].

Cellulose crystallites in the shape of microcrystalline cellulose are now applied extensively in the industrial sector (Figure 15). As past due, novel packages in a diverse field run had been supplied, from sparkling pigments to biomolecular NMR. Within the nanocomposite field, as much as presently, cellulose bristles were applied as a geometrically and essentially well-described display cellulosic filler and no realistic mechanical software replicated this. This is frequently, in particular, ascribed to the duration of the association technique [79].

#### 4.2.1. Automotive Industry

Because cellulose nanomaterials are cheaper relative to different composite reinforcement fibers, it ought to be feasible to use them in extensive-scale structural applications [81]. Ford has stated that it will be able to manufacture many components, from body panels to interior trim; using such material may reduce the weight of its vehicles by 340 kg. Vehicles must be lightweight to meet fuel-efficiency criteria. In the short term, the most likely applications for composite materials will be in applications that already use composite materials, with steel replacement as a secondary application. Cellulose nanofiber (CNF) can be used as a stable, highly reactive raw material for technological applications, with the benefits of being renewable, biologically generated, and biodegradable compared to extraction-based products. For example, reinforcing (bio-) polymers can be used to create promising, ecologically friendly, lightweight construction materials for the automobile industry [39,43].

#### 4.2.2. Food Industry

Nanocellulose has three applications in the food industry: (1) as a stabilizing agent and emulsifier, (2) functional food ingredient, and (3) in food packaging [26]. Salad dressings, creamed toppings, soups, desserts, dips, and a range of other meals have all benefited from nanocellulose as a stabilizing and emulsifying ingredient. Adding 0.25 to 2.00 percent stabilizes nanocellulose to oil/water emulsions containing up to 71.5 percent oil [54].

The percent nanocellulose in a frozen dessert increased the time needed for it to melt and topple. Changing the dessert mix’s composition was unnecessary due to the moderate amount of nanocellulose used because good flavor and texture could be produced, and the frozen dessert’s attributes were not harmed [82].

The food packaging sector produces a lot of synthetic plastics, which are discarded after use and pose a severe environmental risk if they are not recycled [83]. The goal of nanotechnology in food packaging is to extend the shelf life and nutritional content of food while also providing customers with high-quality meals. As a result, several studies have focused on developing biodegradable films and coatings for this industry, which demands using renewable, low-cost, plentiful, and nontoxic biopolymers [84].

Materials with nanocellulose sheets have better mechanical and permeability qualities. The oxygen permeability of nanocellulose films with a thickness of 21 µm was 17 mL $m^{-2}\;{day}^{-1}$, equivalent to polymeric materials such as ethylene vinyl alcohol and polyester-oriented, coated polyvinylidene chloride films of similar thickness.

#### 4.2.3. Papermaking

Papermaking is a long-term industry in which paper recycling has become increasingly important. In Europe, recovered paper accounts for 54 percent of the paper industry’s feedstock, corresponding to a paper recycling rate of 72 percent. However, one of the main obstacles in using secondary fibers is maintaining high-quality levels of paper products in response to client requests. Papermakers must lower manufacturing costs and develop new paper products that approach the tremendous potential of paper as a biodegradable material, such as replacing plastics, to maintain their competitiveness. Nanocelluloses have much potential in this context as strengthening agents, retention system components, and printing quality control aids. Nanocellulose has also shown excellent potential in air permeability controller aids, coating binders, and special additives in technology papers [56].

According to Lindstrom and Aulin, nanocellulose’s more viable and practical uses in the papermaking process are a strength enhancer and barrier applications in food packaging [85]. The first researchers to use micro- and nanocellulose as reinforcing agents in papermaking created them out of wood to improve virgin pulp’s wet and dry strength [6]. Cellulose nanofiber (CNF) improves the pulp’s draining time and water retention by boosting hydrogen bonding, and it has been proved that selecting the correct CNF retention agent and operating circumstances is critical to minimizing or preventing the pulp suspension’s wearability deterioration when CNF is utilized [86].

Moreover, cellulose nanofiber (CNF) may be made by bleaching and homogenizing agro waste pulp without requiring TEMPO oxidation (which is more expensive and has a negative environmental impact than bleaching) and using this CNF boosts strength without compromising drainage [85]. Delgado-Aguilar and Gonzalez found that paper with 1.5 percent by weight CNF had higher stiffness and tensile strength than paper made from beaten pulp with identical WRVs and freeness [87].

### 4.3. Environmental Applications

**Figure 16:**
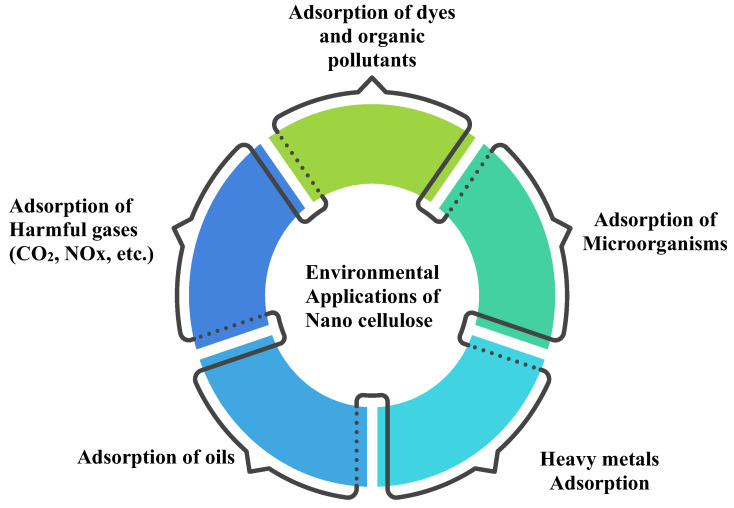
Nanocellulose surface modification techniques classified by pollutant class.

Nanocelluloses are being utilized as a novel class of bio-based adsorbent, beneficial for remedy of the environment due to their high functional capacity, high surface area, and strengthening action [88]. Nanocelluloses can adsorb the most common contaminants, such as dissolved organic pollutants, heavy metals, pigments, oil, and unwanted effluents. To adsorb a specific compound, however, some modification is usually required. A thorough summary of the surface alterations of nanocellulose, the pollutant, has been highlighted in Mahfoudhi and Boufi’s review [89]. Figure 16 illustrates some environmental applications of CNC at a glance.

Finishing and textile dyeing are the primary polluting industries [84]. Dyes, even at small concentrations, influence sunlight penetration and, as a result, reduce marine life’s metabolic oxygen demand, disturbing the food chain downstream. Li and Nandgaonkar created a dye degradation membrane based on the oxidation and subsequent laccase covalent immobilization and TiO_2_. According to the findings, oxidation enhanced the immobilized laccase’s stability. The ideal pH for maximum dye degradation was 56, with an optimum temperature of roughly 40 degrees Celsius. Furthermore, UV irradiation was found to improve dye degradation effectively [84].

### 4.4. Biomedical Applications

**Figure 17:**
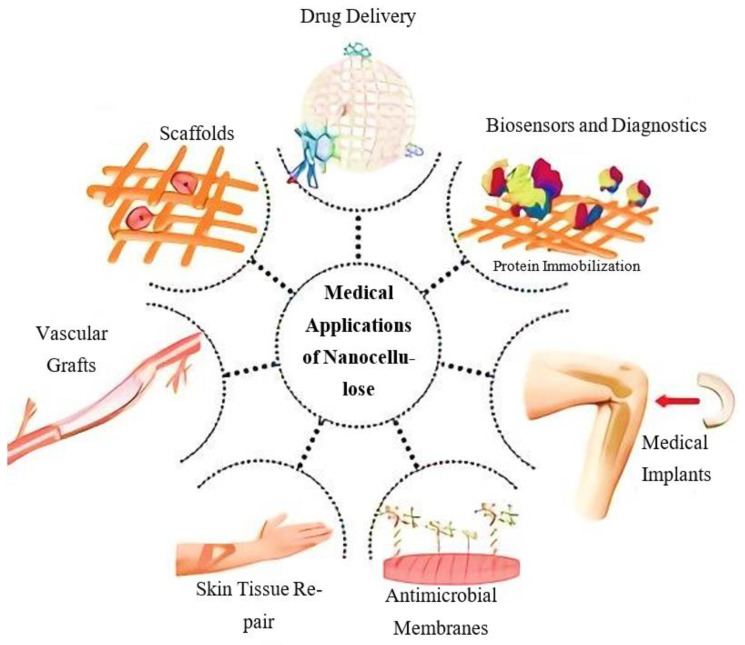
Various applications of CNC in drug delivery and other sectors [101].

Due to their excellent mechanical qualities, nanocomposites have mostly been employed as fillers. They prevent negative tissue responses, are nonhemolytic, and less immunogenic due to their polysaccharide composition and promote cellular connection and tissue growth. The biomedical industry (Figure 17) uses them for skin replacements, drug-releasing systems, gum, nerves, dura mater reconstruction, stent covering, and scaffolds for tissue engineering [90,91,92,93,94]. BNC is a suitable biomaterial for promoting bone regenerative processes. It has been demonstrated that BNC affects bone nodule formation, alkaline phosphatase levels, and osteoblastic cell proliferation.

Recently, a novel concept—the inclusion of goat bone apatite in BNC—was put forth. In this manner, novel bone repair biomaterials that encourage bone cell differentiation and proliferation were created [95].

#### 4.4.1. Drug Delivery and Cancer Therapy

Cellulose nanocrystal is now being researched as a bioactive component in the creation of various delivery of drug systems, especially those that use drugs to cure wounds or illnesses, or dyes to allow for the visualization of carrier trajectory [96,97,98]. Letchford and Jackson looked at the capacity of pure CNC to engage water-soluble antibiotics (tetracycline and doxorubicin) and cationic-ability CNCs to bind these antibiotics to non-ionized and hydrophobic anticancer agents [99,100].

Microspheres, hydrogels, and membranes are the three basic types of CNC-based drug carriers. Controlled delivery systems are a novel technique to control medicinal drug bioavailability. The bioactive treatment is combined into a polymeric framework, and the medication is released from the structure in a predefined manner. The kinetic release can vary depending on the application, and the duration of the drug can range from a few hours to several months [102]. Curcumin-loaded CNC film was developed by Tong et al. as an antimicrobial medication delivery technology in diabetic wound dressing [103]. Because of their rod-shaped morphology and abundance of hydroxyl groups on the surface for functionalization, biocompatible CNC has recently been promising in constructing gene carriers. CNC and gold nanoparticle nanohybrids could combine their respective capabilities for multifunctional cancer therapeutic applications [15,104].

Few studies have focused on CNC–spherical gold nanoparticles (Au NPs) hybrids; polymer brushes on the surface of the CNC were used as capping agents and/or reductants. Hu et al. reported the first-time CNC and Au nanorod (NR) hybrids. As a result, it would be excellent if there were flexible approaches for producing mixtures of CNC and gold nanoparticles with varied morphologies [12,105].

#### 4.4.2. Biosensors and Diagnostics

A one-step bio template technique was used to create Au–BC nanocomposites in aqueous solutions. Excellent biocompatibility, high conductivity, and an ultrafine nanofiber network structure are all shown by this substance. Adhesive amino acid sequences, such as Arg Gly-Asp in extracellular matrix proteins, may help cells connect more effectively [86]. Using CNCs as electrochemical sensors, DNA hybridization has been selectively detected. To find human IgG, peptides such as the HWRGWV peptide may be adsorbed on the CNF. In another investigation, oxidized CNC was coated with silver nanoparticles by TEMPO [35,106].

#### 4.4.3. Repair of Skin Tissue

Nanocellulose membranes might act as an infection barrier, stop fluid loss, relieve pain, make medications easier to apply, and absorb the inflammatory fluids throughout the disease process [107]. They are suited for wound dressings because of characteristics including flexible surface chemistry, anisotropy, optical transparency, high superficial area, high elastic modulus, low thermal expansion coefficient, high water absorption capacity, and biocompatibility. Since it can manage wound exudates and provide a wound with a moist environment that promotes faster injury healing, BC regulates wound dressing. Composite membranes made of BC, gelatin, and alginate allowed NIH/3T3-type cells to proliferate well, demonstrating their potential as a template for skin tissue regeneration. These composites may be utilized for wound dressing since adding chitosan to a tempo-oxidized BC results in a composite with better mechanical characteristics, water holding capacity, and water release rate [48]. Additionally, BC has been utilized in the lateral wall of nose surgery to stop bleeding from the nose, postoperative infections, local discomfort, and clotting [91,108,109].

#### 4.4.4. Vascular Grafts

Artificial bypass implants of polyurethane, polyethylene, and poly(ethylene terephthalate) have been replaced with CNC-based implants. In sheep, pigs, and rats, BC tubes have been utilized to effectively substitute carotid arteries without experiencing any rejection after four weeks. According to research, the CNC-PVA composites’ mechanical characteristics are comparable to those of cardiovascular tissues, including the aorta and heart valve leaflets [110,111].

#### 4.4.5. Cortical Implants

Interfaces made of adaptive cellulose nanoparticles should be rigid enough to be placed readily into the brain but soften when exposed to in vitro conditions. The stiff collagen fibers that make up this nano-cellulosic interface are scattered across a soft fibrillin matrix. For instance, a rubbery ethylene oxide epichlorohydrin copolymer matrix has been effectively used to incorporate CNCs obtained from tunicate marine animals [60,66].

#### 4.4.6. Medical Implants

Medical implants must possess mechanical properties comparable to those of the tissue they replace. Since CNC is non-degradable under physiological settings and offers enduring mechanical qualities and chemical stability, it becomes a promising candidate for use as a medical implant. Dogs undergoing an experimental trochleoplasty were effectively treated with BC. The lack of alveolar bone is a major difficulty in dental applications. BC membranes were evaluated as potential physical barriers to treating bone deformities. After 50 days, nanocellulose accelerated bone repair and produced results similar to the original tissue. PVA/CNC implants did not cause neuron loss 16 weeks after implantation [112].

#### 4.4.7. Antimicrobial Activity

Nanocellulose can be functionalized with antimicrobial agents. A BC film was submerged in a benzalkonium chloride solution in an investigation. Chitin nanocrystal and CNF composites came together to create a 3D network that was bactericidal against E. coli. Mixed gelatin, polyacrylamide, and nanocellulose produce hydrogels with increased toughness [93]. Due to the stromal cells’ ability to integrate into the scaffold, nanomaterials may be employed as corneal tissue scaffolds. Heparin and BC have been cross-linked to inhibit the growth of blood clots. BC has also been used for diabetic foot ulcer sores. Anticoagulant nanocellulose and heparin scaffolds were created for possible application in vascular tissue engineering [58,113,114].

Both bacterial cellulose (BC) and microcrystalline cellulose (MCC) can be involved in the development of carbon-based materials and energy storage devices, but they play different roles and have distinct applications in these areas.

Bacterial Cellulose (BC): Support Material: Bacterial cellulose can serve as a template or support material for the synthesis of carbon-based materials. BC has a unique nanofibrillar structure, which can be used as a scaffold for the growth of carbon nanotubes (CNTs), graphene, or other carbon-based nanomaterials.

Supercapacitors: BC-based composites can be used in supercapacitors and energy-storage devices. The combination of BC with conductive materials such as graphene or carbon nanotubes can enhance the electrical conductivity and capacitance of the composite, making it suitable for energy storage applications [115].

Microcrystalline Cellulose (MCC): Binder and Conductive Additive: MCC is often used as a binder and conductive additive in the fabrication of electrodes for energy-storage devices such as lithium-ion batteries or supercapacitors. It helps hold the active materials together and improve their mechanical integrity.

Carbonization: MCC can be carbonized to produce activated carbon or other carbon materials used in energy-storage applications. Carbonized MCC can serve as an electrode material in supercapacitors or as a component of anodes or cathodes in batteries [115].

## 5. Conclusions and Future Perspectives

From both scientific and economic viewpoints, the usefulness of CNC for various engineering applications is unavoidable, and thus, the CNC has become a promising and exciting area for current and future research and development (R&D). Because of its exceptional physical, chemical, and biological properties nanocellulose holds great potential as an advanced biomaterial. Nanocrystal is combined with various materials to produce various sensors for detecting particles such as gas, chemicals, proteins, electrolytes, glucose etc. This paper provides comprehensive information on how cellulose nanocrystals and functionalized nanocrystals can be used to enhance their performance in terms of physical adsorption, surface grating, and chemical vapor deposition because of the presence of several reactive chemical groups on their surface.

Looking forward, the future of CNCs appears promising. Research into novel applications, further exploration of their biomedical potential, and developing scalable and cost-effective synthesis methods are all exciting avenues for future investigations. CNCs must be prepared cautiously due to their potential risk of aggregation. It should be noted that the redistribution of aggregated CNCs is challenging, resulting in undesirable effects on the composite systems’ sensory and mechanical performances. Due to their increased characteristics, nano-fibrillated cellulose composites are being researched for usage in engineering and medicinal industries. Practical uses of such fillers and the transition to industrial technology necessitate a favorable ratio between predicted and actual costs. There are still considerable scientific and technological obstacles to overcome.

As CNCs continue to gain attention from academia and industry, addressing challenges related to large-scale production, cost-effectiveness, and standardized characterization methods is imperative. Collaborative efforts between researchers, policymakers, and industries will be essential in harnessing the full potential of cellulose nanocrystals.

## Figures and Tables

**Table 1:** Nanocellulose applications in different industrial sectors (low-volume manufacturing).

Industrial Sector	Applications	References
Medicals/ Pharmaceuticals	Drug delivery systems and drug carriers Protein and enzyme recognition and immobilization. Blood vessel replacement Substitutes biomaterials for soft tissue, ligaments, and cartilage replacements. Healing of wounds and skin tissue paper repair Bone tissue healing and regeneration. Purification of blood as hemodialysis membrane	[43,44,45,46]
Cosmetics	Manufacturing of hygiene and absorbent products	[47,48]
Biosensors	Biosensors Manufacturing Manufacturing of flexible electrodes	[49]
Electronic devices	Synthesis of conductive particles Flexible printed electronics Manufacturing of electrical conductors.	[50]
Nano paper	Synthesis of clay Nano paper based on the matrix of CNF and CMF (Cellulose micro fiber)	[35]
Others	Advanced composite materials are applied in aircraft vehicle structures and interiors. Three-dimensional printing, recyclable electronics	[42]

**Table 2:** Nanocellulose applications in different industrial sectors (high-volume manufacturing).

Industrial Sector	Applications	References
Nanocomposites	Matrix reinforcement with polymers Hot compression and extrusion techniques Polymeric matrix types include poly valerolactone (PVL), polylactic acid (PLA), starch, and phosphino-carboxylic acid (PCA)	[49,51,52,53,54]
Plastics	PP and PE reinforcements. Barrier properties and food packaging, mechanical improvements	[39]
Cartoon board and Paper	Bulk and coating addition reinforcing agent for paper and paperboard Print quality for fiber sheets (paper gloss and ink density) Wet-end additives for papermaking [55]	[56,57]
Building	Aerogels for thermal insulation Reinforcement of Concrete formulation As a reinforcement material	[39,49]
Automotive	Light reinforcement agents	[58]
Textiles	Antimicrobial fabrics Conductive fabrics	[5,59]
Food	Used as an additive for low-calorie content Used in food packaging. Ingredient for emulsifying and stabilizing [46]	[60,61]
Environmental	Use in water treatment. Used in air treatment Used in the absorption of pesticides Oil absorption	[17,62,63,64]

**Table 3:** Sensing applications of CNC.

Sensor Type	Description of Composite	Targeted Element	Limit of Detection	Reference
Gas Sensor	PANI/CNC/Cellulose/ TiO_2_/GO	Ammonia	10 ppm	[65]
Chemical sensor	CNC/Lac/Ag/Zno	Formaldehyde	1 ppm	[66]
Protein sensor	GO/CNC/GOx	Glucose	50 ± 10 × 10^−6^ m	[67]
Humidity Sensor	GO/CNC/	Proximity	6mm	[68]
Enzyme sensor	Peptide/CNC	HNE	50 mU mL^−1^	[69]
Ion sensor	Py/CNC	Fe^3+^	10^−3^ × 10^−3^ m	[70]
Glucose sensor	GOx/PPy/CNC/SPE	Glucose	50 ± 10 × 10^−6^ m	[67]
